# Genome-Wide Mapping of Copy Number Variation in Humans: Comparative Analysis of High Resolution Array Platforms

**DOI:** 10.1371/journal.pone.0027859

**Published:** 2011-11-30

**Authors:** Rajini R. Haraksingh, Alexej Abyzov, Mark Gerstein, Alexander E. Urban, Michael Snyder

**Affiliations:** 1 Department of Genetics, Stanford University School of Medicine, Stanford, California, United States of America; 2 Department of Molecular, Cellular and Developmental Biology, Yale University, New Haven, Connecticut, United States of America; 3 Program in Computational Biology and Bioinformatics, Yale University, New Haven, Connecticut, United States of America; 4 Department of Molecular Biophysics and Biochemistry, Yale University, New Haven, Connecticut, United States of America; 5 Department of Computer Science, Yale University, New Haven, Connecticut, United States of America; 6 Department of Psychiatry and Behavioral Sciences, School of Medicine, Stanford University, Stanford, California, United States of America; Tel Aviv University, Israel

## Abstract

Accurate and efficient genome-wide detection of copy number variants (CNVs) is essential for understanding human genomic variation, genome-wide CNV association type studies, cytogenetics research and diagnostics, and independent validation of CNVs identified from sequencing based technologies. Numerous, array-based platforms for CNV detection exist utilizing array Comparative Genome Hybridization (aCGH), Single Nucleotide Polymorphism (SNP) genotyping or both. We have quantitatively assessed the abilities of twelve leading genome-wide CNV detection platforms to accurately detect Gold Standard sets of CNVs in the genome of HapMap CEU sample NA12878, and found significant differences in performance. The technologies analyzed were the NimbleGen 4.2 M, 2.1 M and 3×720 K Whole Genome and CNV focused arrays, the Agilent 1×1 M CGH and High Resolution and 2×400 K CNV and SNP+CGH arrays, the Illumina Human Omni1Quad array and the Affymetrix SNP 6.0 array. The Gold Standards used were a 1000 Genomes Project sequencing-based set of 3997 validated CNVs and an ultra high-resolution aCGH-based set of 756 validated CNVs. We found that sensitivity, total number, size range and breakpoint resolution of CNV calls were highest for CNV focused arrays. Our results are important for cost effective CNV detection and validation for both basic and clinical applications.

## Introduction

Copy Number Variations (CNVs) are a major component of human genomic variation and are thought to be important contributors to phenotypic diversity and human disease [1]. These unbalanced chromosomal rearrangements include duplications, deletions and insertions with respect to a reference genome and lie in the size continuum of genomic variation between small insertions/deletions (indels; 1–1000 bp) and whole chromosomal aneuploidies (multiples of 10 Mbps). Individual CNV events may be benign or pathogenic and can manifest different phenotypes depending on the genomic context of the variant [1], [2]. Multiple studies have mapped CNVs genome-wide in individual genomes using several different technologies e.g. [3]–[11]. Such studies aim to catalog the complement of CNVs within a single genome and to understand their relationships to functional elements, including their implications for normal and disease associated phenotypes. These efforts are curated in databases such as the Database of Genomic Variants (DGV) (http://projects.tcag.ca/variation/). The extent to which CNVs exist in the human genome has not been exhaustively assessed. However, numerous efforts including the recent release of data from the 1000 Genomes Project [12] suggest that the distribution of CNVs in the genome is biased towards multiple hotspots including segmental duplications and away from genes encoding protein complexes and other dosage sensitive genes [3], [8], [13], [14]. CNVs affect a significantly larger fraction of the genome than Single Nucleotide Polymorphims (SNPs) and may encompass several percent of the genome [3], [8].

To date, three main technologies are used for accurate and high-resolution genome-wide CNV mapping [15]; 1) array Comparative Genome Hybridization (aCGH), 2) SNP and SNP plus CGH combination array platforms and 3) 2^nd^ generation sequencing technologies [14], [16]–[18]. Sequencing based technologies are theoretically able to provide base pair resolution of CNV events. However, there remain several obstacles to using only sequencing based methods for CNV mapping. At present, it is still relatively expensive to sequence a genome to the required depth of coverage (currently at least ∼20×) for reliable detection of CNVs greater than 1 kb [3], [12]. Moreover, it is still difficult to execute large-scale genome-wide association type studies due to the limited sample throughput of current 2^nd^ generation DNA sequencing formats. Furthermore, the requisite computational analysis pipelines for identifying CNVs from whole genome sequence data have immense hardware and software requirements. A combination of algorithms is needed to capture a sufficient portion of the CNVs in a sequenced genome [3], and these algorithms are not yet mature enough to be used routinely on the many terabytes of sequencing data generated per genome. In addition, mapping short sequencing reads to non-unique regions of the genome is ambiguous. Over the last 5 years however, increasingly dense and sometimes targeted oligonucleotide arrays for CNV detection have been developed. These have co-evolved with robust and straightforward experimental procedures and with platform specific CNV mining software that can be employed in a reasonably powerful desktop computing workstation. Genome-wide mapping of CNVs by array-based platforms has become a standard approach in phenotype association studies, e.g. [19], [20] clinical cytogenetics e.g. [21] as well as a standard validation tool for CNVs called from whole genome sequence data [3], [8]. As such, array based technologies are likely to be used for CNV mapping for some time.

Currently there are multiple, widely used array platforms that vary in methodology, coverage of the genome, resolution of CNV calls, workflow, and subsequently in ability to accurately and comprehensively detect CNVs. Here we assess the performances of the leading array based platforms for genome-wide CNV detection in the HapMap [22] CEU sample NA12878 against two Gold Standard datasets (GSs). This study differs from previous platform comparisons [23]–[26] in that we have compared the abilities of these platforms to detect known, experimentally validated CNV events genome-wide by taking advantage of the recently released, independent lists of CNVs from the 1000 Genomes Consortium [3], [12] and ultra high-resolution aCGH [8]. These CNV sets are considered the most accurate and complete sets of CNV calls for any genome currently available. The platforms that were compared were the Roche NimbleGen 4.2 M, 2.1 M and 3×720 K whole genome (WG) and CNV focused designs, the Agilent 1×1 M CGH and High Resolution and 2×400 K CNV focused and CGH+SNP designs, the Illumina Omni1Quad and the Affymetrix SNP 6.0 arrays.

We attempted to carry out an unbiased comparative analysis of the practical utilities of these platforms to detect known CNVs in this sample by using data from experiments carried out by the platform manufacturers and the recommended software. We compared the size range, total number, and resolution of the CNV calls from each platform to those of the Gold Standard sets. In addition, we calculated the sensitivity of each platform and found enormous differences. We also showed that each platform detects additional CNVs that are not included in the stringent Gold Standard sets. Finally, for the platforms that require control samples, we investigated the value of using control DNA from a single sequenced individual compared with that from a pool of individuals. We found that the use of a single genome control led to higher performance. These studies are important for maximizing value from the many thousands of CNV probing experiments performed each year.

## Results

### Genome-wide CNV detection by twelve platforms

We compared the genome-wide CNV detection abilities of twelve different array-based platforms from four different manufacturers (summarized in Table 1): Roche NimbleGen (hereafter referred to as NimbleGen) 4.2 M, 2.1 M and 3×720 K whole genome (WG) and CNV focused designs, Agilent 1×1 M CGH and High Resolution designs and 2×400 K CNV focused and CGH+SNP designs, Illumina Human Omni1Quad and Affymetrix SNP 6.0 arrays. These platforms differ in a number of features including total number of probes and their spacing; these and other experimental details are also included in Table 1. Additionally, we compared two different conditions (single genome control versus pool of genomes) for the two NimbleGen 2.1 M designs. Thus a total of 14 different platform CNV sets are compared in this paper.

**Table 1:** Summary of CNV detection platforms and experiments.

Platform	Total Features	Median Probe Spacing	Data Source	Technical Replicates	Control Sample	Analysis	Total CNVs Replicate 1, Replicate 2	Total CNV Overlap with 1000G GS	Total CNV Overlap with 42 M GS
***aCGH Platforms***									
Roche NimbleGen Human CGH 4.2 M WG Tiling Array	4,200,000 *(50–75mers)*	284 bp	NimbleGen	2	NA10851	NimbleScan 2.6 (converted to HG18 using LiftOver from UCSC)	425,465	83, 87	115, 112
Roche NimbleGen Human CNV 4.2 M Array	4,200,000 *(50–75mers)*	284 bp	Service Provider	2	NA10851	NimbleScan 2.6 (converted to HG18 using LiftOver from UCSC)	1926, 1883	408, 386	310, 275
Roche NimbleGen Human CGH 2.1 M WG Tiling Array	2,100,000 *(60mers)*	1.1 kb	NimbleGen	2	NA10851	NimbleScan 2.6	498, 482	73, 78	98, 95
			Snyder	2	Pool of 7 females (Promega)		229, 103	47, 18	64, 22
Roche NimbleGen Human CNV 2.1 M Array	2,100,000 *(50–75mers)*	1.2 kb (backbone)	NimbleGen	2	NA10851	NimbleScan 2.6	1939, 1981	286, 299	251, 253
			Snyder	2	Pool of 7 females (Promega)		481, 605	66, 63	21, 24
Roche NimbleGen Human CGH 3×720 K WG Tiling Array	720,000 *(60mers)*	2.5 kb	NimbleGen	2	NA10851	NimbleScan 2.6	206, 187	24, 26	29, 29
Roche NimbleGen Human CNV 3×720 K Array	720,000 *(50–75mers)*	4.8 kb (backbone)	NimbleGen	2	NA10851	NimbleScan 2.6	819, 897	160, 179	166, 178
Agilent SurePrint G3 Human CGH Microarray, 1×1 M	963,029 *(60mers)*	2.1 kb overall (1.8 kb in RefSeq Genes)	Service Provider	2	NA10851	AGW6.5	825, 879	57, 59	94, 76
Agilent SurePrint G3 Human High Resolution Microarray, 1×1 M	963,331 *(60mers)*	2.6 kb	Service Provider	2	NA10851	AGW6.5 (converted to HG18 using LiftOver from UCSC)	1566, 1604	91, 90	114, 107
Agilent SurePrint G3 Human CNV Microarray, 2×400 K	442,892 *(60mers)*	1 kb in CNVs	Service Provider	2	NA10851	AGW6.5 (converted to HG18 using LiftOver from UCSC)	1055, 1002	49, 128	158, 184
***aCGH+SNP Platforms***									
Agilent SurePrint G3 Human CGH+SNP Microarray, 2×400 K	292,097 (CGH) *(60mers)* 119,091 (SNP)	7.2 kb overall (4.5 kb in Refseq genes)	Agilent	2	NA12891	AGW6.5 (converted to HG18 using LiftOver from UCSC)	120, 126	4, 5	15, 16
Illumina Human Omni1-Quad BeadChip	1,140,419 (SNP)	1.2 kb	Service Provider	2	N/A	GenomeStudio 2010.2 (converted to HG18 using LiftOver from UCSC)	251, 267	62, 68	122, 122
Affymetrix SNP 6.0	946,000 (CGH) *(25mers)* 906,000 (SNP)	CN 2.2 kb SNP 1.3 kb Overall 0.7 kb	Mc. Carroll et al.	1	N/A	Birdseed	162	49	67

DNA from the same sample, HapMap [22] CEU sample NA12878, which has been extensively characterized by the 1000 Genomes Project [12], was hybridized to each platform. In most cases, hybridizations were performed either by the company that manufactures the arrays or by a service provider; however, one set of NimbleGen experiments was performed by us, and the Affymetrix SNP 6.0 array data was already published. Two technical replicates were performed for all experiments. For most cases the control DNA was comprised of the individual HapMap CEU sample NA10851, which matched published results used in our Gold Standard set (see below). However, for one Agilent platform the well-characterized HapMap CEU NA12891 DNA was used. Analyses of the raw data for generating the CNV lists were carried out using the manufacturers' software and recommended parameter settings as described in the methods.

**Figure 1:**
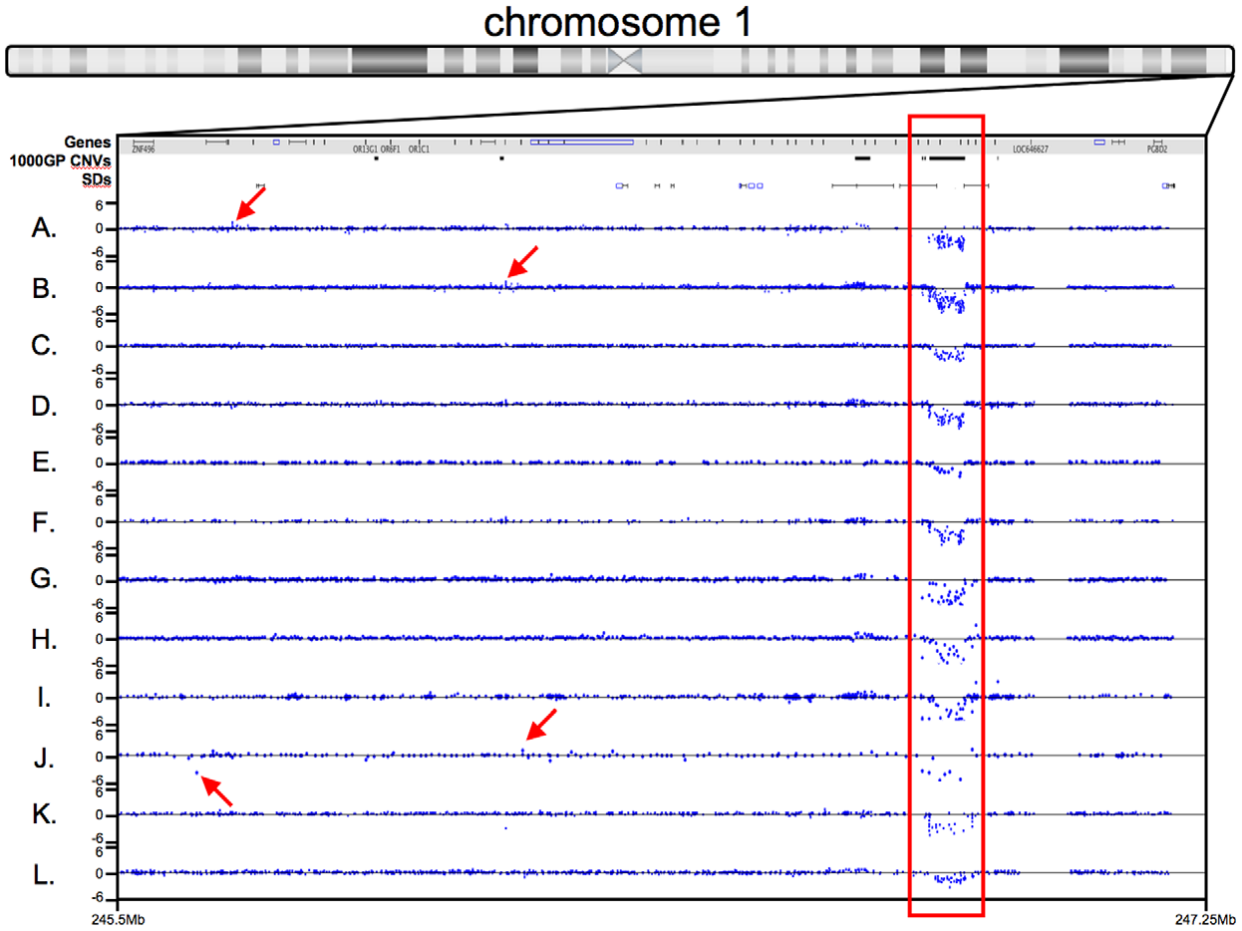
Detection of a known 57 kb CNV on chromosome 1q44 by twelve different array platforms. Depicted are the log_2_ ratios of NA12878 to the control genome and locations along the chromosome for each probe (blue dot) in this region. Gene, 1000 Genomes Project Gold Standard CNVs (1000GP CNVs) and Segmental Duplication (SD) tracks are shown. The CNV highlighted in the red box was called by both replicates for all twelve platforms using the manufacturer-recommended software and parameters. Red arrows indicate examples of additional array-specific CNV calls. A. NimbleGen 4.2 M Whole Genome, B. NimbleGen 4.2 M CNV, C. NimbleGen 2.1 M Whole Genome, D. NimbleGen 2.1 M CNV, E. NimbleGen 3×720 K Whole Genome, F. NimbleGen 3×720 K CNV, G. Agilent 1×1 M CGH, H. Agilent 1×1 M High Resolution, I. Agilent 2×400 K CNV, J. Agilent 2×400 K CGH+SNP, K. Illumina Human Omni1Quad, L. Affymetrix SNP 6.0.

**Figure 2:**
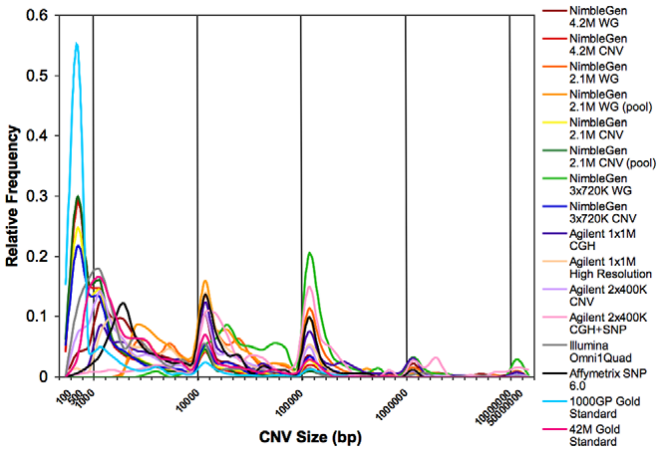
Size distributions of CNV call sets. Data include CNVs from all replicates of the fourteen different experiments listed in table 1 (twelve different platforms and two different conditions for NimbleGen 2.1 M arrays). The size distributions of the two Gold Standards are overlaid for reference. The apparent frequency spikes are results of the changing bin size.

The different platforms called from 103 (NimbleGen 2.1 M Whole Genome against pool) to 1981 (NimbleGen 2.1 M CNV) total CNVs (Table S1) in the genome of NA12878. Only one independently validated CNV (57 kb) was detected by all platforms (Figure 1) and many other CNVs were unique to individual or distinct sets of platforms. The CNV that was detected by all platforms falls within an Olfactory Receptor gene cluster, a known CNV hotspot [27]. This common CNV has been reported by several studies using diverse methods e.g. [6], [8], [28], [29]. The 1000 Genomes Project reports this CNV as two distinct events, a 57 kb deletion on one allele and a 53 kb deletion on the other allele entirely contained within the bounds of the 57 kb deletion. However, aCGH cannot differentiate these two alleles and calls the CNV as a single event. Interestingly the breakpoints lie in consecutive segmental duplications indicating that this event is likely to be mediated by non-allelic homologous recombination of these segmental duplications [30]. In addition, the probe tracks show that the probe densities for the twelve arrays are highly variable. The size distributions of the CNVs called by each platform are shown in Fig. 2. More than 50% of events called by the NimbleGen CNV focused arrays are less than 2 kb in size, with up to 36%<500 bp; this result is expected since these arrays have high tiling density in known CNV regions and the largest number of probes. More than 50% of the Illumina Omni1Quad calls are <3 kb whereas ∼50% of the Agilent 2×400 K CNV calls are <6 kb. More than 80% of CNVs called by the NimbleGen 4.2 M and 3×720 K WG designs and Agilent 1×1 M CGH and 2×400 K CGH+SNP arrays are 1–500 Kb in size. More than 90% of CNVs called by the NimbleGen 2.1 M WG, Agilent 1×1 M High Resolution and the Affymetrix SNP 6.0 arrays are in the 1–500 kb size range. All the arrays have a similar frequency distribution of CNV calls in the larger size ranges. The size distributions of the Gold Standard CNV sets (described below) are shown for reference. More than 50% of the 1000GP Gold Standard CNVs are less than 500 bp (75%<1 kb) while more than 50% of the 42 M Gold Standard CNVs are 500 bp-3 kb in size.

### The Different Platforms Can Detect Many Known CNVs

To determine the relative performances of the different platforms we compared the CNV sets obtained from each array to Gold Standard datasets. Two independent sets of Gold Standard CNVs (GS CNVs) were assembled: one set was from the 1000 Genomes Pilot Project sequence data [3], [12] and the other from a study that used ultra-high resolution aCGH [8]. The 1000 Genomes Project Gold Standard (1000GP GS) is the set of experimentally validated sequence-based CNV calls found in NA12878 by the 1000 Genomes Project July 2010 data release. The final set of 3997 unique CNVs in the genome of NA12878 was determined at base pair resolution using Read Depth, Split Read and Paired-end analysis of 2nd generation sequencing data. Each CNV in the final set was validated by aCGH (NimbleGen or Agilent custom arrays) or by PCR. The validated CNV calls from this sequence data are thought to be the most accurate set of calls for this sample to date [3]. We also used a second gold standard, the Roche NimbleGen 42 million aCGH Gold Standard (42 M GS). This is the list of CNVs found in the genome of NA12878 by aCGH using a set of 20 NimbleGen arrays containing ∼42 million long oligo probes (50–75 bp) in total tiling the non-repetitive portion of the genome (median spacing ∼56 bp) and by using NA10851 as a control (the same control that we used). This set consists of 756 unique CNVs >450 bp in size and containing a minimum of 10 consecutive probes per CNV. Validation of a ubset of calls was carried out by qPCR and by aCGH on a custom designed Agilent CNV genotyping array [8]. Because the resolution of this aCGH experiment far exceeds that of the platforms that were analyzed in this paper, this validated set could be used as a gold standard. We realize that the use of a NimbleGen specific gold standard for comparing NimbleGen platforms to those of other manufacturers may be of concern. However, the trends observed in our comparison generally agree regardless of the gold standard used, so we believe any such potential bias is negligible. These two different gold standard sets are complementary since they were derived from differing technologies that have different abilities to detect CNVs (summarized in Table 2). In total, only 255 (6%) 1000GP GS CNVs (corresponding to 205 (27%) 42 M GS CNVs) occur in both sets using the 50% reciprocal overlapping criterion described below.

**Table 2:** Summary of possible relationships between reference, experimental control and test genomes and the resulting abilities of different methods to detect CNVs in the test genome (with respect to the reference genome).

Genome relationships at a given locus	Test CNV detection by aCGH against Control	Test CNV in 1000GP Gold Standard (against Reference Genome)	Test CNV in 42 M aCGH Gold Standard (against Control)
Test≠Control = Reference	yes	yes	yes
Test≠Control≠Reference	yes	yes	yes
Test = Control≠Reference	no	yes (but those >500 bp were removed from our GS for fairer comparison)	no
Test = Control = Reference	no	no	no
Test = Reference≠Control	yes	no	yes

Copy Number Variants in one genome can only be defined with respect to a reference genome. Comparisons to different reference genomes will produce different sets of CNVs for the same test genome. Sequencing based methods directly compare the test genome to the reference genome. For array-based methods however, this comparison is indirect as an experimental control genome is also required. The relationship between the reference genome and this control genome determines which CNVs in the test sample (with respect to the reference genome) are detected.

We assessed the performance of each array by several metrics including the number of GS CNVs detected, their size, their breakpoint resolution (i.e. the platform CNV size relative to defined GS breakpoints), the sensitivity of each platform and the number of non-GS CNVs called by each platform. To determine the validity of a platform CNV call we used an extension of the criterion established by the 1000 Genomes Project for counting two different CNV calls as the same event [3]: a platform CNV is considered validated if either it overlaps a single GS CNV by at least 50% reciprocally, or there exists a set of GS CNVs such that each member of the set overlaps the platform call by at least 50% of the GS CNV size and the total number of base pairs of the platform call that overlap some member of this set of GS CNVs is at least 50% of the size of the platform call. Henceforth, any further mention of overlap will imply these criteria unless otherwise stated.

**Figure 3:**
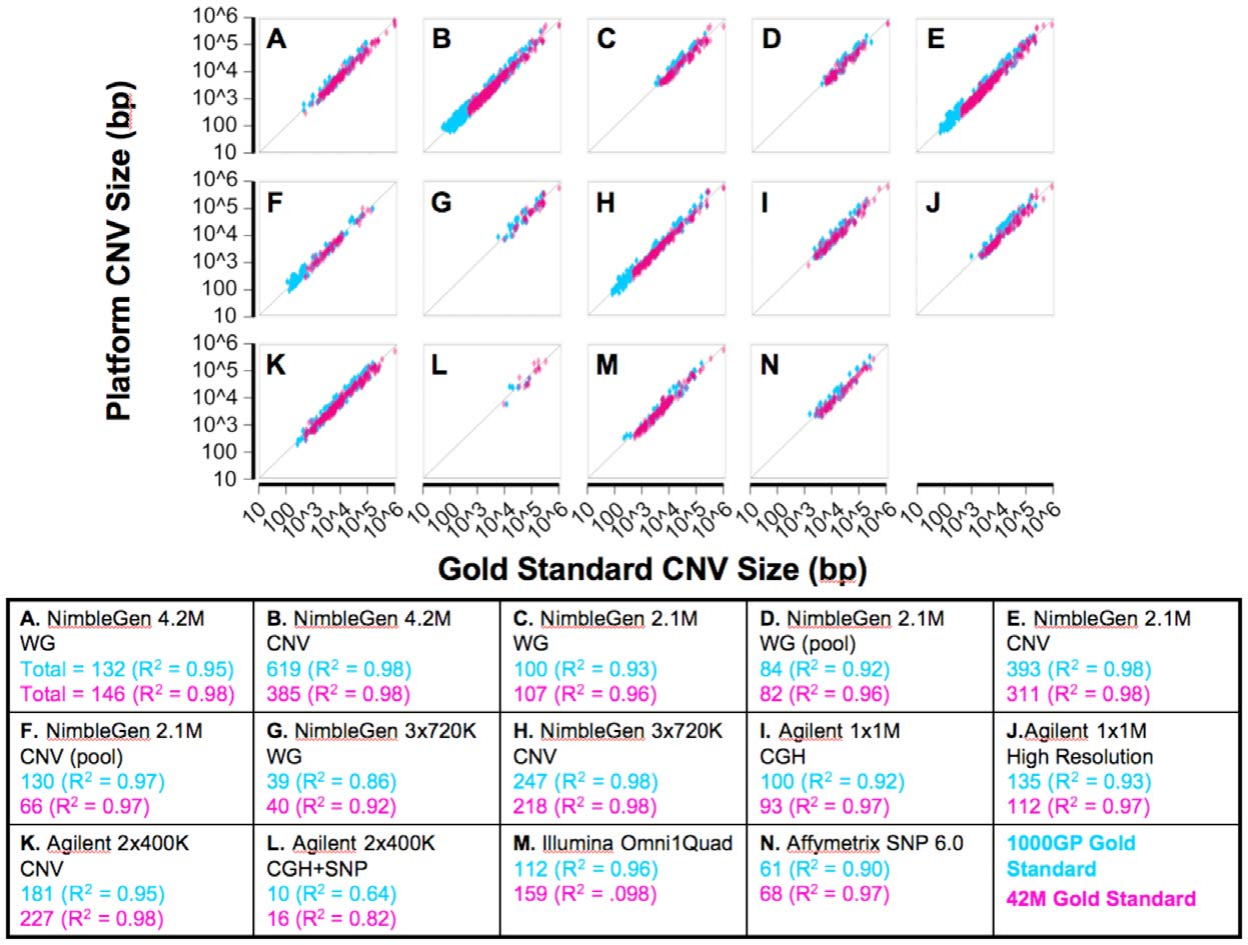
Resolution of array platform CNV calls for known Gold Standard CNVs. Each data point plots the size of a platform CNV versus the size of the corresponding Gold Standard CNV that individually overlaps the platform call by 50% reciprocally. Events overlapping the 1000GP GS CNVs are shown in blue. Events overlapping the 42 M GS CNVs are shown in pink. Total numbers of data points and R^2^ values are indicated in the table below. Data is an aggregate of all events from two technical replicates for each platform except the Affymetrix SNP 6.0. The size distribution and total number of platform CNVs that overlap Gold Standard CNVs in this way are also clearly visible.

Each platform identified many GS CNVs. A summary of the sizes of the GS CNVs detected by each platform is shown in Figure 3. Overall, the greatest absolute number of GS CNVs detected by each platform was found by the NimbleGen 4.2 M CNV (619 1000GP GS CNVs, 385 42 M GS CNVs) followed in order from most GS CNVs detected to least by the NimbleGen 2.1 M CNV, 3×720 K CNV, Agilent 2×400 K CNV, Agilent 1×1 M High Resolution, NimbleGen 4.2 M WG, Illumina Omni1Quad, NimbleGen 2.1 M WG, Agilent 1×1 M CGH, Affymetrix SNP 6.0, NimbleGen 3×720 K WG and Agilent 2×400 K CGH+SNP (10 1000GP GS CNVs, 16 42 M GS CNVs) platforms. This order is largely preserved regardless of the gold standard used.

For CNVs greater than 10 kb, 10–71 1000GP GS CNVs and 11–88 42 M GS CNVs were detected. The largest numbers of 1000GP GS and 42 M GS CNVs >10 kb in size were detected by the NimbleGen 4.2 M CNV and the Agilent 2×400 K CNV arrays respectively while the smallest numbers were detected by the Agilent 2×400 K CGH+SNP array. In addition to the larger CNVs, most of the platforms detected much smaller CNVs albeit with reduced sensitivity. The NimbleGen 4.2 M CNV array detected the largest numbers of GS CNVs <10 kb in size (548 1000GP GS CNVs and 322 42 M GS CNVs). The smallest GS CNVs are detected by the NimbleGen CNV focused designs followed by the Agilent 2×400 K CNV and the NimbleGen 4.2 M WG designs, the Illumina OmniQuad, the Agilent 1×1 M designs and the Affymetrix SNP 6.0, the NimbleGen 2.1 M WG array, the NimbelGen 3×730 K WG array and finally the Agilent 2×400 K CGH+SNP array. The platforms with the highest probe densities in known CNV regions are the ones that detect the largest numbers of GS CNVs and also the smallest GS CNVs. At the extremes, the NimbleGen CNV focused arrays can detect GS CNVs <100 bp, whereas the smallest GS CNV detected by the Agilent CGH+SNP array is on the order of 10 kb.

For each platform, the sizes of the platform calls were generally similar to those of the overlapping GS CNVs. These results indicate that when a CNV is detected, its breakpoints are reasonably accurate.

### Sensitivities of the Different Platforms

**Figure 4:**
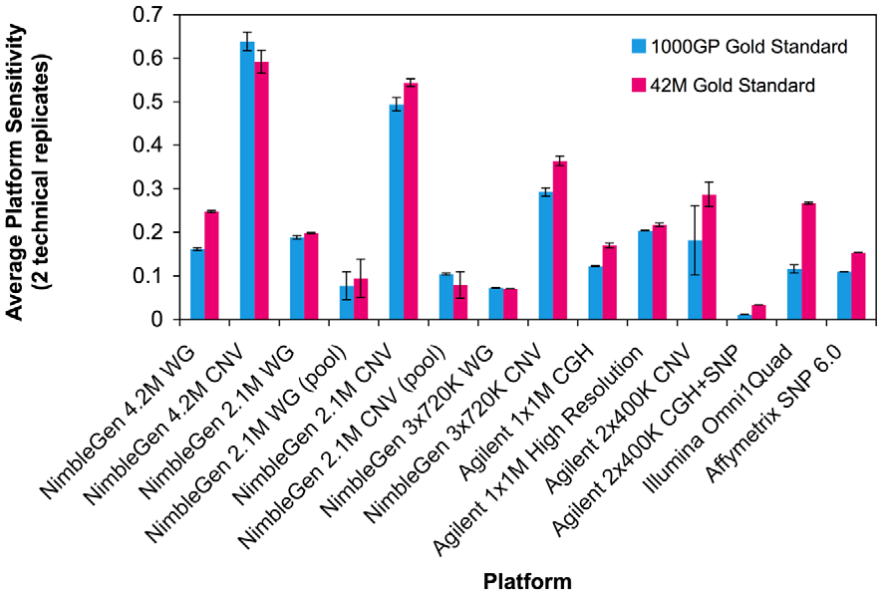
Array sensitivities to detecting Gold Standard CNVs. Depicted are the average corrected platform sensitivities based on two technical replicates for each platform, except the Affymetrix SNP 6.0. Corrected sensitivities were calculated using only those Gold Standard CNVs that were detectable by at least one platform. Blue bars show sensitivity to the detectable 1000GP GS and pink bars show sensitivity to the detectable 42 M GS.

**Figure 5:**
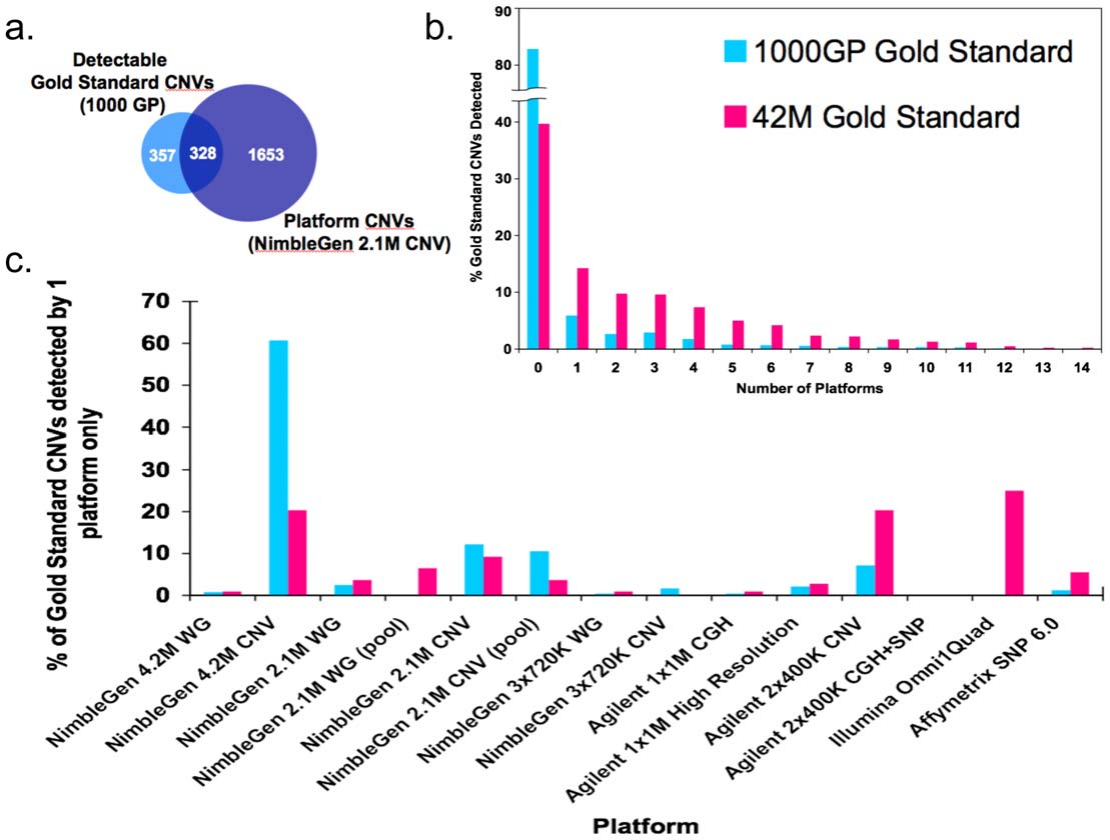
Array detectable Gold Standard CNVs. (a) Venn diagram showing overlap of CNV calls from a single platform (NimbleGen 2.1 M CNV) with the detectable 1000 Genomes Gold Standard. (b) The percentage of Gold Standard CNVs that were detected by at least one technical replicate of each platform is shown for all platforms (including twelve different platforms and two conditions for the NimbleGen 2.1 M arrays). Blue bars indicated percentage of 1000GP GS CNVs and pink bars indicate percentage of 42 M GS CNVs. (c) Platform distribution of all Gold Standard CNVs that are detected by only one platform.

The average sensitivity to each gold standard was calculated for each platform (Figure 4). Sensitivities ranged from 0.01 (Agilent 2×400 K CGH+SNP) to 0.64 (NimbleGen 4.2 M CNV). The platform CNVs that overlapped detectable GS CNVs by 50% reciprocally were counted as true positives (Figure 5a). Since the overlap criteria do not necessarily imply a one-to-one overlap between a platform CNV and a GS CNV, the overlapping CNVs were counted with respect to the GS CNVs. The NimbleGen 4.2 M CNV array clearly stands out with the highest sensitivity followed by the NimbleGen 2.1 M CNV and 3×720 K CNV arrays while the Affymetrix SNP 6.0, NimbleGen 3×720 K WG and the Agilent SNP+CGH arrays have the lowest sensitivities regardless of the GS used. The NimbleGen 4.2 M and 2.1 M WG designs, the Agilent 1×1 M and 2×400 K CNV designs, and the Illumina Omni1Quad all have comparable sensitivities with some platforms performing slightly better than others depending on the GS used. Raw sensitivities for all the platforms were very low (Figure S1). In the process of generating a maximally unbiased report of sensitivity, we noted that a large subset of GS CNVs (>80% of 1000GP GS and ∼40% of 42MGS) were not detectable by any platform (Figure 5b). We therefore defined detectable sets of GS CNVs to be the subsets of GS CNVs detected by at least one platform. We used these detectable GS CNV sets to report corrected sensitivities here. Most GS CNVs are detected by relatively few platforms and only two CNVs from each gold standard are found in all 14 platform CNV lists (the two GS CNVs are detected as one single platform CNV). We further assessed the platform distribution of the GS CNVs detected only by one platform (Figure 5c). The CNV focused platforms detect >80% of these platform-specific 1000GP GS CNVs. The NimbleGen 4.2 M CNV, Agilent 2×400 K CNV and Illumina Omni1Quad arrays detect the majority of the platform-specific 42 M GS CNVs (>20% each).

### Platform Distribution of Detectable Gold Standard CNVs

**Figure 6:**
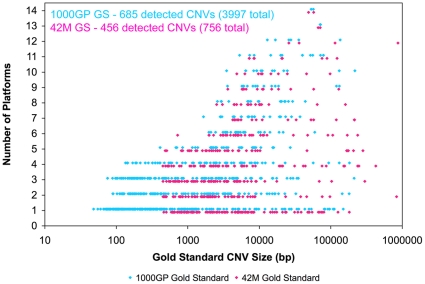
Number of array platforms detecting each Gold Standard CNV by size. Each data point represents the number of platforms that detect a certain Gold Standard CNV by at least one technical replicate using our overlap criteria versus the size of the Gold Standard CNV. Only detectable Gold Standard CNVs are shown. Blue points correspond to 1000GP GS CNVs and pink points correspond to 42 M GS CNVs.

We analyzed in detail the number of platforms that can identify each detectable GS CNV as a function of size using our overlap criteria (Figure 6). In general, as shown in the figure the larger events are detected by more platforms. Events detected by 8 or more platforms are often >2.5 kb in size. However, there are still many large GS CNVs that are missed by most platforms; 50% of detectable 1000GP GS CNVs and 44% of detectable 42 M GS CNVs greater than 50 kb are found by only 4 or fewer platforms (for the 1000GP GS the majority of these were found by they NimbleGen 2.1 designs and the 3×720 K WG array; for the 42 M GS the majority of these were found by Agilent CNV array). The smaller CNVs are detected by only a few platforms. 98% of detectable 1000GP GS CNVs and 89% of detectable 42 M GS CNVs less than 2 kb are found by four or fewer platforms. Interestingly, several (275 1000GP GS and 12 42 M GS) GS CNVs less than 500 bp are detectable by commercial arrays. In summary, there are many CNVs both large and small that are detected by only a limited number of platforms.

### All Platforms Detect Many Non-Gold Standard CNVs

Across all platforms, large percentages (70–96% for 1000GP GS and 52–93% for 42 M GS) of platform calls are not in the gold standards sets (Figure S2). At the extremes, 52% of the Illumina Omni1Quad CNVs did not overlap with the 42 M GS whereas 96% of the Agilent 2×400 K CGH+SNP CNVs did not overlap with the 1000GP GS by our criteria. These platform calls may fall into several categories; some may be false positives, some may be undetectable by sequencing based CNV methods, some may be true but not included in the gold standards for various reasons including inability to validate using PCR or custom aCGH or due to the very strict requirements for inclusion into the 42 M GS.

### More CNVs are Detected Using a Single Genome Control versus a Pooled Control

We expect to call at least twice as many CNVs from an aCGH experiment using a single individual genome as a control versus a pool of genomes. This is because the call set from the aCGH experiment against a single control comprises both test (NA12878) and control (NA10851) CNVs without any way of differentiating from which genome an individual CNV signal originated. However, in an aCGH experiment using a pool of genomes as a control, the signals of many rare CNVs that are present in individual genomes of the control pool are expected to be too weak to be detected. Thus, the call set from such an experiment is likely only to contain CNVs from the test genome (NA12878). This trend was observed for the NimbleGen 2.1 M whole genome and CNV focused arrays. For the NimbleGen 2.1 M whole genome array, approximately three times as many calls were made when the NA 10851 DNA was used as a control (490 average CNVs) relative to the pooled reference (166 average CNVs). For the NimbleGen 2.1 M CNV focused array ∼3.6 times as many CNVs were called using the single control (1960 average CNVs) relative to the pool (543 average CNVs) (Table 1). Thus, more CNVs can be identified using a single individual genome as the control in an aCGH experiment as opposed to using a pool of genomes as the control.

As expected, more CNVs from experiments using a single control overlapped with both GS CNV sets than those from experiments using a pooled control. For a given platform, the size distributions and resolutions of the validated calls are comparable regardless of the control used (Figure 3c–e). In addition, we calculated higher sensitivities to both gold standards for experiments using a single individual as a control than for those using a pooled control (bars 3–6 of Figure 4 and Figure S1). The sensitivity of the NimbleGen 2.1 M whole genome array was twice as high when a single genome was used as a control compared to when a pool of genomes was used. The sensitivity of the NimbleGen 2.1 M CNV focused array was 5–7 times higher when a single genome was used as a control than when a pool of genomes was used. These results indicate that aCGH experiments using an individual sequenced control sample may provide a valuable means for maximal CNV detection.

## Discussion

CNVs form an important class of human genetic variants. Understanding their functional impacts requires accurate, comprehensive and efficient genome-wide mapping [1]. This study evaluated the abilities of the current generation of array-based CNV detection platforms to detect known CNVs genome-wide.

There are fundamental differences between CGH, CGH+SNP and SNP only platforms. These include differences in methodologies (single versus dual channel experiments and scanning signal acquisition) and in physical attributes of the arrays (probe type and oligomer length) [15]. For example, SNP and combination array CNV calls are based on single channel experiments where the control and test DNA samples are hybridized to different arrays leading to less reliable log_2_ ratios of the signal intensity of test DNA to control DNA than for aCGH. However, SNP arrays also combine SNP genotype data (the proportion of minor alleles to total alleles at a locus) to complement log_2_ ratio data for CNV calling. aCGH platforms rely exclusively on log_2_ ratios for CNV calling. Because of such differences we have tried to assess only the practical utilities of the various platforms for CNV calling. Consequently, we have compared only the final lists of CNV calls from each platform for a particular genome (NA12878) based on the experimental and data analysis protocols recommended by each array manufacturer. In our comparison we assume that all platforms contain probes that are optimized to be maximally informative at their specific loci. This assumption allows interpretation of our results based on probe distributions and probe numbers regardless of probe type (CGH or SNP). We note that many different algorithms are available for extracting CNV data from all of the platforms reviewed here. Algorithm and parameter effects can result in highly variable CNV call sets from the same raw array data. Such algorithm effects are reviewed elsewhere [26] and are beyond the scope of this manuscript. Therefore we have chosen to assess only the practical utilities of these platforms which we believe include the use of the best manufacturer recommended algorithms and parameters, as these are the most likely analyses that will be carried out by users.

Most array platforms attempt to balance unbiased whole genome novel CNV discovery and focused genotyping in known CNV regions under the restraint of fixed probe numbers by employing intelligent probe distributions. For SNP arrays (Affymetrix and Illumina), probe distributions are restricted by the non-uniform availability of informative SNPs throughout the genome [9]. It is especially difficult to find informative SNPs to sample repetitive regions, where many CNVs occur [14], [15]. In contrast, for CGH arrays, probes can be selected with a more even genome-wide distribution in general, and with higher density in certain regions as desired. Sometimes, aCGH probes can be designed to unique stretches of sequence in repetitive regions even in the absence of SNPs. Consequently most SNP arrays are now complemented by additional CGH probes [9]. We found that probe distribution greatly affects the performance of a platform.

By all metrics including total number of platform calls, size range of calls, resolution of calls and platform sensitivity, the NimbleGen and Agilent CNV focused arrays were the top performers. Notably, for both NimbleGen and Agilent arrays, the CNV focused designs had more than four times as many total calls and sensitivities that were at least two times higher compared to the corresponding unbiased designs with the same numbers of probes. Additionally, these arrays (along with the Illumina Omni1Quad array) detected the largest size range of CNV events from ∼500 bp-1 Mbp. Furthermore, the NimbleGen 3×720 K CNV and Agilent 2×400 K CNV designs consistently performed better than arrays with even more probes such as the NimbleGen 4.2 M WG, 2.1 M WG, Agilent 1×1 M designs, Illumina Omni1Quad and the Affymetrix SNP 6.0. Additionally when comparing the Agilent 1×1 M gene focused CGH array against the evenly tiled High Resolution array we see that probe distribution affects the performance of the arrays whereby the evenly tiled array outperforms the gene-centric design albeit by only small margin. We conclude that probe distribution appears to be the dominant factor affecting array performance in this study. CNV focused designs outperform more evenly tiled designs that in turn outperform gene-focused designs in detecting CNVs genome-wide.

It is expected that arrays containing more probes will detect larger numbers of CNVs genome-wide and call breakpoints that are generally closer to the true endpoints CNVs than arrays with fewer probes. This is because CNVs can only be detected in genomic regions for which probes exist. When comparing arrays with similar probe distributions (whole genome or CNV focused), we do find the expected trend of arrays with more probes performing better than those with fewer; the NimbleGen 4.2 M whole genome and CNV designs perform better than the corresponding 2.1 M designs which perform better than the 3×720 K designs which perform better than the corresponding Agilent 2×400 K designs. However, the total number of probes does not appear to be a sufficient predictor of CNV detection ability. Rather, for the current generation of arrays, probe selection seems to be the most important factor in optimizing CNV detection.

It is expected that a paucity of probes in certain regions will lead to the inability of an array to make robust CNV calls in those regions. We suspect that this is the primary reason why such large fractions of the GS CNV sets are not detected by any of the platforms (∼80% of the 1000GP GS CNVs and ∼40% of the 42 M GS CNVs are not detected). There is a large percentage of CNVs in the 1000GP GS (22% Alus, 1% LINEs) that are too repetitive to be tiled by aCGH probes or to contain informative SNPs, and will consequently not be detected by any of the tested platforms. No Alu in the 1000GP GS was detectable by any array platform. The 42 M GS does not contain any SINE or LINE repeats. Furthermore, 75% of CNVs in the 1000GP GS and 18% CNVs in the 42 M GS are less than one 1 kb in size. We do not expect most platforms to detect CNVs <1 kb in size even in unique sequence. This is because most of the array platforms do not contain enough probes to provide the required probe density to detect such small events genome-wide. The CNV focused arrays sometimes contain the required probe density for detecting certain small CNVs without compromising backbone probe density. These platforms also detect the largest subsets of the GS CNVs that are detected by a single platform only, for the same reason. Thus, while the numerical values are specific to this study the trends of array performance reported here are as expected.

Our performance metrics do not take into account the additional genome-wide SNP data provided by several of these platforms (Agilent GCH+SNP, Illumina, Affymetrix). It will depend on a given study design to determine whether additional SNP information is worth the seemingly reduced sensitivity of CNV detection by these platforms. The recent availability of platforms such as the Illumina Omni2.5 M array with 2.5 million SNP and CGH probes may increase the ability of these platforms to detect both CNVs and SNPs. However, optimized analyses for detecting CNVs from these arrays are not yet available, and thus they were excluded from this study. Ultimately, as whole genome sequencing costs continue to drop and analysis methods for extracting CNV (and Structural Variation in general), SNP and mobile element insertion data become more developed, the utility of arrays will be replaced by the availability of the entire spectrum of genomic variation at base pair resolution [12]. In the near future however, because of both cost considerations and the orthogonal information provided relative to sequencing, array methods are likely to continue to be used for large-scale CNV studies in biological and medical research, validation of sequencing-based CNV data [3] and for routine clinical diagnostics [21].

In this study we specifically analyzed known CNVs in a European sample relative to European controls. This undoubtedly led to biases in CNV detection especially since some of the arrays (NimbleGen and Agilent CNV focused arrays, Illumina Omni1Quad and Affymetrix SNP 6.0 arrays) were designed to detect CNVs found in the 1000 Genomes Project pilot trios. These trios include the Caucasian trio of which NA12878 is the daughter and a Yoruban trio [3], [12]. Thus, the results obtained may differ when analyzing different combinations of ethnicities.

All the platforms called large numbers of non-GS CNVs. While some of these observations are certainly false positives, it is unlikely that ∼80% of all calls on any single array are false. More plausibly, many of these array-detected non-GS CNVs may be true CNVs that do not occur in the gold standards for several reasons. Both gold standards were assembled using extremely strict requirements. The 1000GP GS comprised only experimentally validated calls. However, validation by PCR and aCGH was attempted only on a subset of the total calls [3]. Additionally, the 1000GP calls were made using three different analyses; Read Depth, Paired-end and Split Read analysis. Each of these methods has specific limitations. Read depth will not call CNVs that occur in highly repetitive elements (SINEs and LINEs). Split read and Paired-end analyses produce less confident calls using the short reads (36 bp) of the 1000 Genomes pilot data as these are less likely to map uniquely to the reference genome. Therefore we expect and observe little overlap of the calls from these three methods as noted by the 1000 Genome Project [3]. However, the CNVs used in our Gold Standard are only those that were found by at least two out of three computational methods and experimentally validated; it is likely that many real CNVs exist but were detected by one method only, not validated or entirely missed. For inclusion into the 42 M GS, CNVs were required to contain at least 10 probes in sequence [8]. However, there may be many CNVs containing less than 10 probes. We conclude that there may be many true platform calls that do not occur in the gold standards due to the stringent inclusion criteria. Another reason why such large numbers of platform calls did not overlap with GS CNVs could be due to the overlap criterion that was used. We required 50% reciprocal overlap to count two CNV calls as the same. However, the breakpoints of CNVs obtained from array-based methods can be up to several kb from the true breakpoints. Hence there may be true events called by the platforms that are not counted as valid in our analyses because the overlap may be less than 50%.

Lastly, we addressed the important issue as to whether a single genome is a more informative control than a pool of genomes for aCGH. An aCGH experiment using a single genome control will miss all the CNVs with respect to the reference that are exactly the same in the test and control samples. This issue can be resolved by using a single control that is well characterized for CNVs by some orthogonal method. The rationale for using a pool of individual genomes as the control is to eliminate this loss due to the expectation that at most loci, the copy number of most of genomes of the pool will be the same as that of the reference. Thus, the effects of rare CNVs in individual genomes of the pool are diluted. However, for polymorphic loci in which a significant subset of the genomes of the pool contain the same CNV as the test sample, that CNV may not be called in the test sample as the signal would be dampened. For cancer samples or other heterogeneous tissue, this problem can be exacerbated against a normal control as the heterogeneity of the tissue leads to an even more dampened signal. We found that using a single individual genome as a control provided more CNV calls and higher sensitivities than using a pool of genomes as a control.

In conclusion we have shown that under the recommended manufacturers' experimental and analytical conditions, there is enormous variability in performance of the current generation of widely used commercial CNV detection arrays. The arrays best able to detect known CNVs in a well characterized European sample are those that contain dense probe tiling in known CNV regions while not compromising the backbone tiling density of the rest of the genome.

## Methods

### Sample Selection

All individual samples analyzed in this study were chosen from the 1000 Genomes Project [12] and previously from the International HapMap Project [22]. The test sample, NA12878, was chosen because of extensive prior genomic characterization including ∼42× sequence coverage in the 1000 Genomes Project and ultra-high resolution array Comparative Genome Hybridization [8]. NA12878 is a Utah resident of European Ancestry (CEU) and is the daughter in one of the two trios sequenced at high coverage in the 1000 Genomes Pilot Project. The control sample NA10851 was also chosen from extensive genomic characterization including its use as the control in ultra-high resolution aCGH and as a 1000 Genomes Project low coverage sample [8], [12]. NA10851 is a male of European Ancestry from Utah (CEU). NA12891, the father of NA12878, was used as the control for the Agilent CGH+SNP 2×400 K array. Genomic DNA for these samples was obtained from the Coriell Institute for Medical Research. An additional control, a pool of 7 females (Promega, Cat # G1521) was chosen because it is a commercially available, reproducible pool of individual genomes.

### Gold Standard CNV sets for NA12878

Two complementary Gold Standard sets of CNVs found in the genome of NA12878 were used in this study. These are the 1000 Genomes Project Gold Standard and the Roche NimbleGen 42 million aCGH Gold Standard described below.

#### 1000 Genomes Project Gold Standard (1000GP GS)

The 1000 Genomes Project Gold Standard is the set of validated CNVs found in the genome of NA12878 by the 1000 Genomes Project [3], [12] during the recently completed pilot phase of the effort. The set consists of sequence-based NA12878 CNV calls (using the inner confidence interval coordinates) from the July 2010 data release that were validated by aCGH (NimbleGen or Agilent custom arrays) or PCR. Deletion data and duplication data were obtained from:

ftp://ftp-trace.ncbi.nih.gov/1000genomes/ftp/pilot_data/release/2010_07/trio/sv/

ftp://ftp-trace.ncbi.nih.gov/1000genomes/ftp/pilot_data/release/2010_07/low_coverage/sv/

ftp://ftp-trace.ncbi.nih.gov/1000genomes/ftp/pilot_data/paper_data_sets/companion_papers/mapping_structural_variation/.

Only those calls that were made in the genome of NA12878 and contained the word ‘validated’ at least once in the validation column were included. The set was further pruned by removing all calls where the start coordinate was larger than the end coordinate. In addition, NA10851 CNVs occurring in this set were removed so as not to confound the findings. NA10851 CNVs were called using CNVnator [17] from the low coverage 1000 Genomes sequence data. The final set consists of 3997 unique CNVs. The 1000 Genomes Project utilized a variety of methods including Read Depth, Split Read and Paired-end mapping to call high quality, base pair resolution CNVs from next generation sequencing data on NA12878. The CNV calls from this sequence data are thought to be the most accurate set of calls for this sample to date [3].

#### Roche NimbleGen 42 million aCGH Gold Standard (42 M GS)

The Roche NimbleGen 42 million aCGH Gold Standard is the set of published CNVs found in NA12878 using NA10851 as a control on a set of 20 Roche NimbleGen arrays each containing ∼2.1 million long-oligo probes (50–75 bp) tiling the non-repetitive portion of the genome (median spacing ∼56 bp). The parameters were set to detect CNVs greater than 500 bp [8]. The total set consists of 756 unique CNVs. The resolution of this aCGH experiment exceeds that of the platforms being compared in this paper, and hence the calls from this data are likely to be more accurate in size and breakpoint resolution than those from the platforms being compared here. Thus, this set was used as a Gold Standard. In order to call CNVs from this data, strict criteria were used including a minimum of 10 consecutive probes to call an event. A subset of calls was validated by qPCR and by aCGH on a custom designed Agilent CNV genotyping array based on the initial CNV call set [8].

### Generation of Platform CNV sets for NA12878

NA12878 CNV call sets from twelve different array-based platforms from four different manufacturers were compiled using raw data obtained from either the manufacturer or a service provider followed by data processing using the manufacturers' recommended software and parameters. The platforms used were the NimbleGen 4.2 M, 2.1 M and 3×720 K whole genome and CNV focused designs, Agilent 1×1 M CGH and High Resolution designs and 2×400 K CNV focused and CGH+SNP designs, Illumina Human Omni1Quad and Affymetrix SNP 6.0 arrays (summarized in Table 1). Details of how the individual call sets were compiled are described below.

#### Roche NimbleGen NA12878 CNV call sets

Raw data from aCGH experiments performed on six different platforms were obtained from Roche NimbleGen, Inc. (Madison, WI 53719, USA). The platforms used were the NimbleGen 4.2 M, 2.1 M and the 3×720 K whole genome and CNV focused arrays. All experiments used NA12878 DNA as the test sample and NA10851 DNA as the control sample. In addition, aCGH experiments were performed by us using the manufacturer's protocol with NA12878 DNA as the test sample and a pool of female genomic DNA as the control on the 2.1 M whole genome and CNV focused designs. In brief, NA12878 DNA was labeled with cy3 and the control pool DNA was labeled with cy5. Equal amounts of the test and control DNA were hybridized to the arrays for 72 hours. The arrays were washed and scanned in an ozone free environment using a Roche MS200 scanner. Images were analyzed using NimbleScan 2.6 software (Roche NimbleGen, Inc., Madison, WI 53719, USA). Two technical replicates of all experiments were performed. In order to obtain final CNV call sets from raw data for each experiment, segtable files were generated using NimbleScan 2.6 with default settings (min segment difference = 0.2, min number of probes per segment = 2). Segments with −0.25<Log R<0.25 were removed. For the 4.2 M and 2.1 M designs, segments with <5 probes per segment were also removed. The 4.2 M designs are based on HG19 coordinates. These CNV coordinates were converted to HG18 using the UCSC LiftOver tool [31]. The coordinates of 425/435 and 465/474 CNV calls from two technical replicates of the WG array and 1926/1956 and 1883/1912 CNV calls from two technical replicates of the CNV array were successfully converted. The remaining CNV coordinates comprised the final call sets for these arrays.

#### Agilent NA12878 CNV call sets

Raw data from aCGH experiments carried out on the Agilent 1×1 M CGH and High Resolution designs and 2×400 K CNV focused design using NA12878 DNA as the test and NA10851 DNA as the control were obtained from service providers. Raw data from hybridizations and scannings of the Agilent 2×400 K CGH+SNP array using NA12878 DNA as the test and NA12891 DNA as the control were obtained from Agilent Technologies (Santa Clara CA 95051, United States). Final CNV call sets were obtained by generating Interval Based Reports in the Agilent Genomic Workbench 6.5 software package (Agilent Technologies, Santa Clara CA 95051, United States) using default settings. Chromosomal coordinates for the resulting CNV calls are based on HG19 (except for the 1×1 M CGH design). The CNV coordinates were converted to HG18 using the UCSC LiftOver tool [31]. The coordinates of 1566/1615 and 1604/1651 CNV calls from two technical replicates of the 1×1 M High Resolution array were successfully converted. The coordinates of 1055/1094 and 1002/1045 CNV calls from two technical replicates of the CNV focused 2×400 K array were successfully converted. The coordinates of 120/124 and 126/129 CNV calls from two technical replicates of the CGH+SNP 2×400 K array were successfully converted. The remaining CNVs comprised the final call sets for these platforms. Technical replicates for all 2×400 K designs are from the same field of separate arrays.

#### Illumina NA12878 CNV call sets

Raw data for two technical replicates of NA12878 DNA hybridized to the Illumina Human Omni1Quad as per the manufacturer's protocol were obtained from a service provider. The data were analyzed using Genome Studio 2010.2 software (Illumina, Inc., San Diego, CA 92121 USA) in which SNP clustering and genotyping were performed, B allele frequencies (the proportion of minor (‘B’) alleles to total (‘A’ and ‘B’) alleles at a locus) were calculated and log_2_ ratios were extracted. CNV analysis was carried out using the CNVpartition 2.4.4 algorithm within Genome Studio using default parameters (Confidence Threshold = 35). The obtained CNV lists comprised coordinates based on HG19. These coordinates were converted to HG18 using the UCSC LiftOver tool [31]. The coordinates of 251/259 and 267/277 calls from the two technical replicates were successfully converted and comprised the final lists of NA12878 CNVs from this platform.

#### Affymetrix NA12878 CNV call set

The set of CNVs found in the genome of NA12878 by the Affymetrix SNP 6.0 array was obtained from the supplement of published data [9]. No further processing was done on this set before including it in the comparative analyses.

## Supporting Information

Figure S1 **Array sensitivities to detecting Gold Standard CNVs.** Depicted are the average raw platform sensitivities based on two technical replicates for each platform, except the Affymetrix SNP 6.0. Blue bars show sensitivity to the 1000GP GS and pink bars show sensitivity to the 42 M GS.
(TIF)

Figure S2 **Non-Gold Standard Platform CNV discovery rate.** Depicted are the proportions of individual Platform CNV call sets that do not meet the 50% reciprocal overlapping criteria with Gold Standard CNVs. Calculations are based on two technical replicates for each platform except the Affymetrix SNP 6.0. Blue bars show values with respect to the 1000GP GS and pink bars show values with respect to the 42 M GS.
(TIF)

Table S1 **Platform Specific Raw Data.** This table contains the following information for each replicate of all microarray platforms; total number of CNV calls, total GS overlapping CNVs, sensitivity calculations (raw and corrected) and false discovery rate calculations.
(XLS)

## References

[1] Stankiewicz P, Lupski JR (2010). Structural variation in the human genome and its role in disease.. Annu Rev Med.

[2] Hurles ME, Dermitzakis ET, Tyler-Smith C (2008). The functional impact of structural variation in humans.. Trends Genet.

[3] Mills RE, Walter K, Stewart C, Handsaker RE, Chen K (2011). Mapping copy number variation by population-scale genome sequencing.. Nature.

[4] Pang AW, MacDonald JR, Pinto D, Wei J, Rafiq MA (2010). Towards a comprehensive structural variation map of an individual human genome.. Genome Biol.

[5] Craddock N, Hurles ME, Cardin N, Pearson RD, Plagnol V (2010). Genome-wide association study of CNVs in 16,000 cases of eight common diseases and 3,000 shared controls.. Nature.

[6] Altshuler DM, Gibbs RA, Peltonen L, Dermitzakis E, Schaffner SF (2010). Integrating common and rare genetic variation in diverse human populations.. Nature.

[7] Park H, Kim JI, Ju YS, Gokcumen O, Mills RE (2010). Discovery of common Asian copy number variants using integrated high-resolution array CGH and massively parallel DNA sequencing.. Nat Genet.

[8] Conrad DF, Pinto D, Redon R, Feuk L, Gokcumen O (2010). Origins and functional impact of copy number variation in the human genome.. Nature.

[9] McCarroll SA, Kuruvilla FG, Korn JM, Cawley S, Nemesh J (2008). Integrated detection and population-genetic analysis of SNPs and copy number variation.. Nat Genet.

[10] Kidd JM, Cooper GM, Donahue WF, Hayden HS, Sampas N (2008). Mapping and sequencing of structural variation from eight human genomes.. Nature.

[11] Redon R, Ishikawa S, Fitch KR, Feuk L, Perry GH (2006). Global variation in copy number in the human genome.. Nature.

[12] Durbin RM, Abecasis GR, Altshuler DL, Auton A, Brooks LD (2010). A map of human genome variation from population-scale sequencing.. Nature.

[13] Schuster-Böckler B, Conrad D, Bateman A (2010). Dosage sensitivity shapes the evolution of copy-number varied regions.. PLoS One.

[14] Korbel JO, Urban AE, Affourtit JP, Godwin B, Grubert F (2007). Paired-end mapping reveals extensive structural variation in the human genome.. Science.

[15] Alkan C, Coe BP, Eichler EE (2011). Genome structural variation discovery and genotyping.. Nat Rev Genet.

[16] Abyzov A, Gerstein M (2011). AGE: defining breakpoints of genomic structural variants at single-nucleotide resolution, through optimal alignments with gap excision.. Bioinformatics.

[17] Abyzov A, Urban AE, Snyder M, Gerstein M (2011). CNVnator: An approach to discover, genotype, and characterize typical and atypical CNVs from family and population genome sequencing.. Genome Res.

[18] Medvedev P, Stanciu M, Brudno M (2009). Computational methods for discovering structural variation with next-generation sequencing.. Nat Methods.

[19] Jarick I, Vogel CI, Scherag S, Schäfer H, Hebebrand J (2011). Novel common copy number variation for early onset extreme obesity on chromosome 11q11 identified by a genome-wide analysis.. Hum Mol Genet.

[20] Ionita-Laza I, Rogers AJ, Lange C, Raby BA, Lee C (2009). Genetic association analysis of copy-number variation (CNV) in human disease pathogenesis.. Genomics.

[21] Miller DT, Adam MP, Aradhya S, Biesecker LG, Brothman AR (2010). Consensus statement: chromosomal microarray is a first-tier clinical diagnostic test for individuals with developmental disabilities or congenital anomalies.. Am J Hum Genet.

[22] Consortium IH (2003). The International HapMap Project.. Nature.

[23] Tucker T, Montpetit A, Chai D, Chan S, Chénier S (2011). Comparison of genome-wide array genomic hybridization platforms for the detection of copy number variants in idiopathic mental retardation.. BMC Med Genomics.

[24] Oldridge DA, Banerjee S, Setlur SR, Sboner A, Demichelis F (2010). Optimizing copy number variation analysis using genome-wide short sequence oligonucleotide arrays.. Nucleic Acids Res.

[25] Curtis C, Lynch AG, Dunning MJ, Spiteri I, Marioni JC (2009). The pitfalls of platform comparison: DNA copy number array technologies assessed.. BMC Genomics.

[26] Pinto D, Darvishi K, Shi X, Rajan D, Rigler D (2011). Comprehensive assessment of array-based platforms and calling algorithms for detection of copy number variants.. Nat Biotechnol.

[27] Hasin Y, Olender T, Khen M, Gonzaga-Jauregui C, Kim PM (2008). High-resolution copy-number variation map reflects human olfactory receptor diversity and evolution.. PLoS Genet.

[28] Matsuzaki H, Wang PH, Hu J, Rava R, Fu GK (2009). High resolution discovery and confirmation of copy number variants in 90 Yoruba Nigerians.. Genome Biol.

[29] Perry GH, Ben-Dor A, Tsalenko A, Sampas N, Rodriguez-Revenga L (2008). The fine-scale and complex architecture of human copy-number variation.. Am J Hum Genet.

[30] Hastings PJ, Lupski JR, Rosenberg SM, Ira G (2009). Mechanisms of change in gene copy number.. Nat Rev Genet.

[31] Hinrichs AS, Karolchik D, Baertsch R, Barber GP, Bejerano G (2006). The UCSC Genome Browser Database: update 2006.. Nucleic Acids Res.

