# Supplementary figures and images for: Genome-Wide Mapping of Copy Number Variation in Humans: Comparative Analysis of High Resolution Array Platforms
Source: PLoS One. 2011 Nov 30;6(11):e27859. doi: 10.1371/journal.pone.0027859 (PMC3227574; doi:10.1371/journal.pone.0027859)

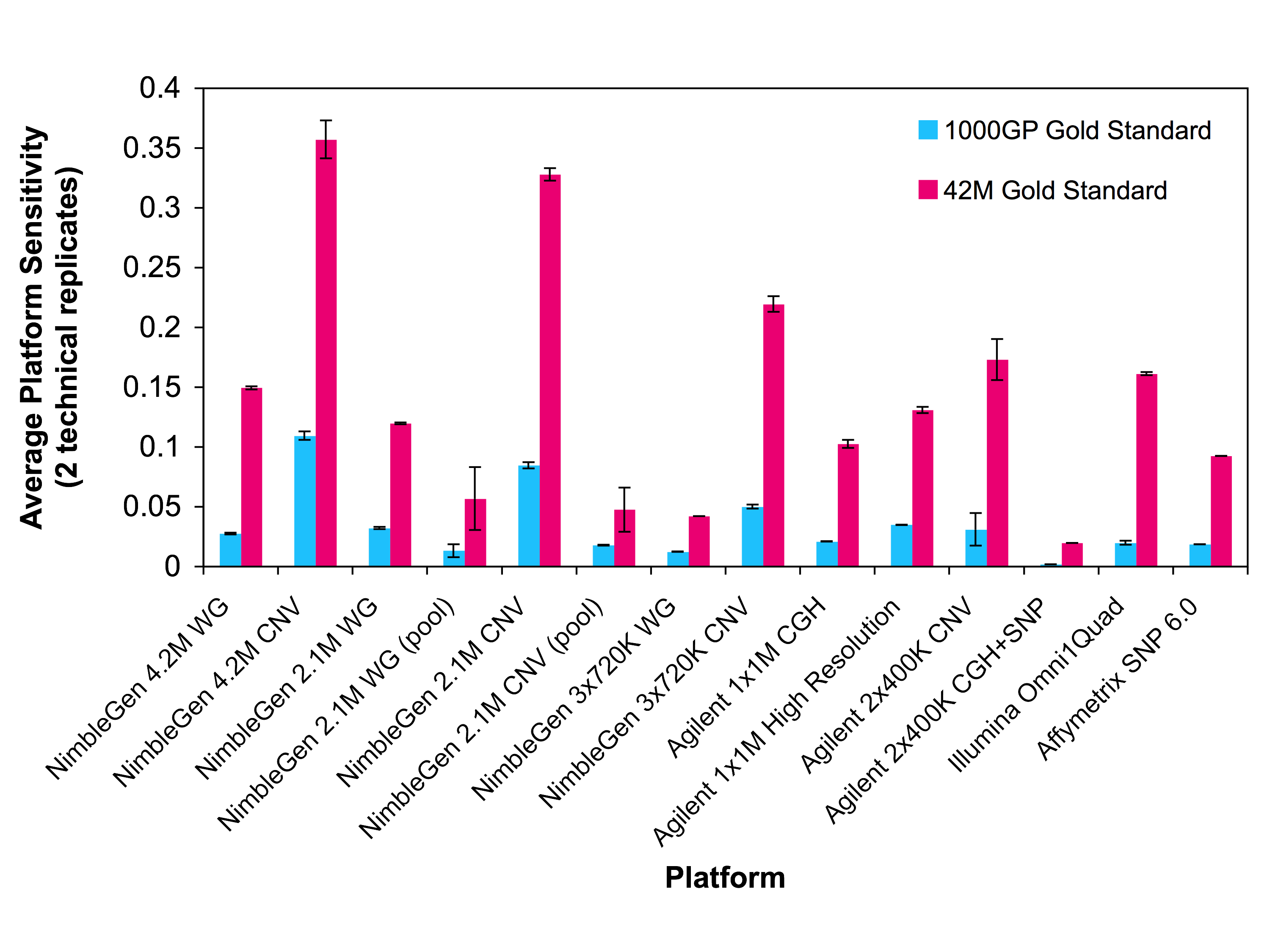

Supplement: Figure S1 — Array sensitivities to detecting Gold Standard CNVs. Depicted are the average raw platform sensitivities based on two technical replicates for each platform, except the Affymetrix SNP 6.0. Blue bars show sensitivity to the 1000GP GS and pink bars show sensitivity to the 42 M GS. (TIF) [file pone.0027859.s001.tif]

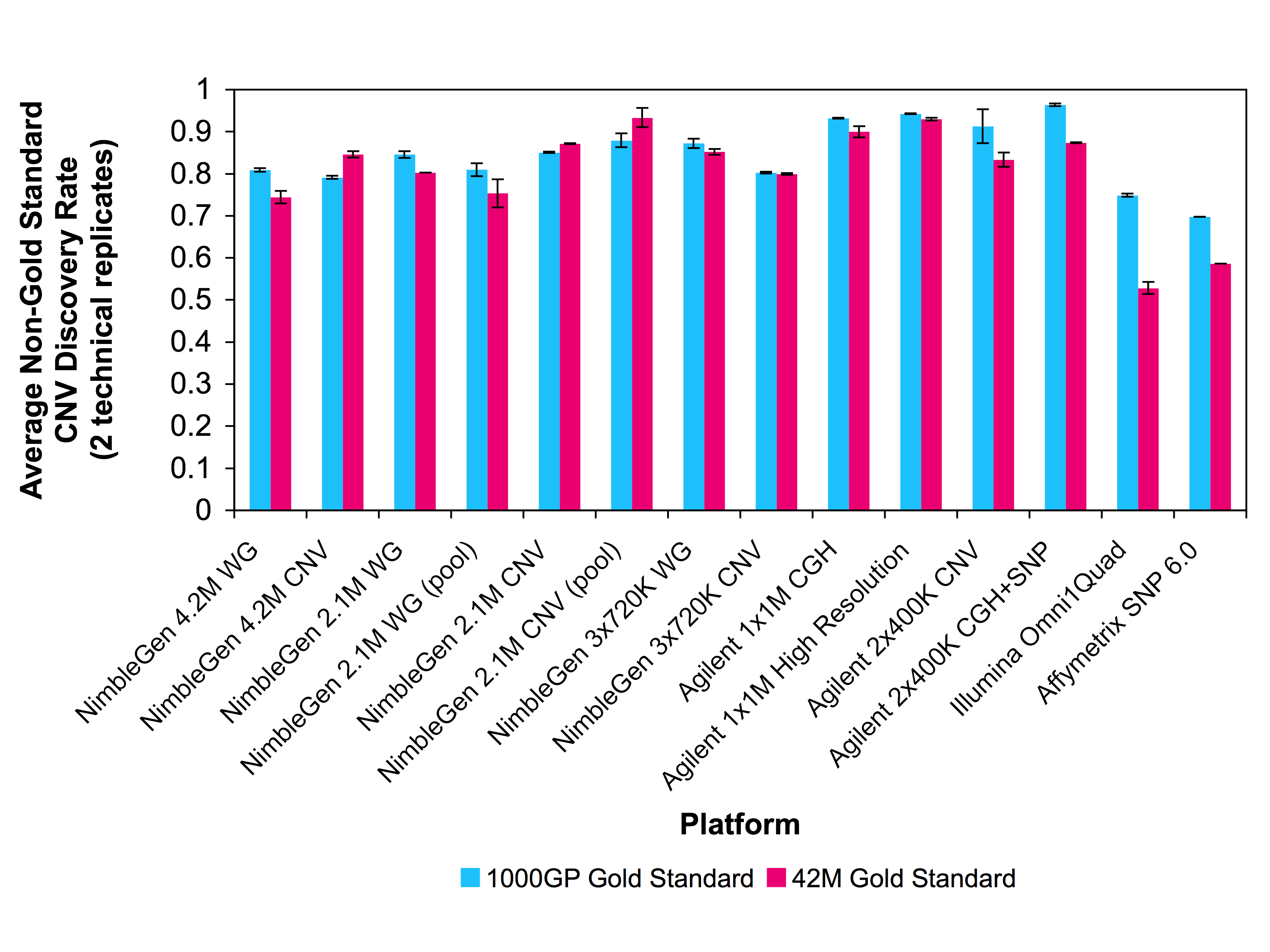

Supplement: Figure S2 — Non-Gold Standard Platform CNV discovery rate. Depicted are the proportions of individual Platform CNV call sets that do not meet the 50% reciprocal overlapping criteria with Gold Standard CNVs. Calculations are based on two technical replicates for each platform except the Affymetrix SNP 6.0. Blue bars show values with respect to the 1000GP GS and pink bars show values with respect to the 42 M GS. (TIF) [file pone.0027859.s002.tif]
